# Risk factors and prediction model for osteonecrosis of the femoral head in female systemic lupus erythematosus

**DOI:** 10.3389/fimmu.2024.1381035

**Published:** 2024-08-21

**Authors:** Wenbo Xu, Lihe Wang, Pengbo Shi, Linfeng Liu, Wenxin Zhang

**Affiliations:** ^1^ College of Orthopedics and Traumatology, Henan University of Chinese Medicine, Zhengzhou, China; ^2^ Department of Orthopedics and Traumatology, the First Affiliated Hospital of Henan University of Chinese Medicine, Zhengzhou, China

**Keywords:** risk factors, osteonecrosis of the femoral head, systemic lupus erythematosus, menstrual abnormalities, nomogram, lasso regression analysis, female

## Abstract

**Background:**

Osteonecrosis of the femoral head (ONFH) is a severe complication of systemic lupus erythematosus (SLE) and occurs more frequently in SLE patients than in other autoimmune diseases, which can influence patients’ life quality. The objective of this research was to analyze risk factors for the occurrence of ONFH in female SLE patients, construct and validate a risk nomogram model.

**Methods:**

Clinical records of SLE patients who fulfilled the 1997 American College of Rheumatology SLE classification criteria were retrospectively analyzed. The Least absolute shrinkage and selection operator (LASSO) regression and multivariate logistic regression analysis were used to summarize the independent risk factors of ONFH in female SLE patients, which were used to develop a nomogram. The predictive performance of the nomogram was assessed using the receiver characteristic (ROC) curve, calibration curves and decision curve analysis (DCA).

**Results:**

793 female SLE patients were ultimately included in this study, of which 87 patients (10.9%) developed ONFH. Ten independent risk factors including disease duration, respiratory involvement, menstrual abnormalities, Sjögren's syndrome, osteoporosis, anti-RNP, mycophenolate mofetil, cyclophosphamide, biologics, and the largest daily glucocorticoid (GC) were identified to construct the nomogram. The area under the ROC curve of the nomogram model was 0.826 (*95% CI*: 0.780–0.872) and its calibration for forecasting the occurrence of ONFH was good (*χ^2^* = 5.589, *P* = 0.693). DCA showed that the use of nomogram prediction model had certain application in clinical practice when the threshold was 0.05 to 0.95. In subgroup analysis, we found that the risk of ONFH was significantly increased in age at SLE onset of ≤ 50 years old, largest daily GC dose of ≥50 mg and the therapy of GC combined with immunosuppressant patients with menstrual abnormalities.

**Conclusion:**

Menstrual abnormalities were the first time reported for the risk factors of ONFH in female SLE patients, which remind that clinicians should pay more attention on female SLE patients with menstrual abnormalities and take early interventions to prevent or slow the progression of ONFH. Besides, the nomogram prediction model could provide an insightful and applicable tool for physicians to predict the risk of ONFH.

## 1 Introduction

Systemic lupus erythematosus (SLE) is a systemic autoimmune disease with a complex and diverse clinical presentations (1). Osteonecrosis (ON), characterized by ischemia-induced necrosis of bone cells, is a common and serious complication of SLE, with a prevalence rate of 1.7% to 52% (2). Compared to the knee, elbow and shoulder joints, the femoral head is the joint most commonly affected by ON (ONFH) in SLE patients (3). ONFH commonly begins with obscure symptoms and can progress to severe pain at the site of the lesion and movement limitation, requiring joint arthroplasty in the late stage (4), which not only seriously affects the quality of life in SLE patients, but also imposes a heavy economic burden on SLE patients. Therefore, early ONFH risk assessment with SLE can contribute to early screening, prevention, and intervention with ONFH.

The etiology of SLE-ONFH is multi-factorial. Over the years, many studies have concluded that glucocorticoid (GC) use is the predominant risk factor for ONFH, especially short-term, high-dose GC (5, 6). Besides, current studies have indicated that the use of immunosuppressants, SLE associated with clinical factors including disease duration, osteoporosis, Sjögren's syndrome, respiratory involvement and autoimmunity antibodies, are also related with the risk of ONFH (7–9). Genetics is also contributed to the risk of SLE-ONFH (10). A clinical study (11) found an intronic WIPF1 variant that could independently increase the occurrence of ON risk in SLE patients. However, current studies on risk factors for SLE-ONFH suffer from small sample sizes, inconsistent results, and a lack of comprehensive and reliable prediction models to guide clinical prevention of SLE-ONFH. Besides, women are more susceptible to develop SLE than men (12), especially women of childbearing age, which can cause multiple organ damage in its early stages. Among organ damage, menstrual abnormalities including abnormal menstrual cycle frequency, irregular menstrual cycles, light menstrual bleeding, heavy menstrual bleeding, and dysmenorrhea are common in SLE patients (13–15). In clinical practice, when we took the menstrual history of SLE patients, we found an interesting phenomenon that female patients with SLE-ONFH often had menstrual abnormalities. We suspected that female SLE patients with menstrual abnormalities may be more likely to occur ONFH. However, there are no reports on the relationship between ONFH and menstrual abnormalities in SLE patients. Exploring whether menstrual abnormalities are one of the risk factors is worthwhile.

Therefore, the aim of our study was to identify the independent risk factors for ONFH in female SLE patients by investigating conventional clinical indicators and conduct a preliminary exploration of whether menstrual abnormalities are one of risk factors for SLE-ONFH, which would be more helpful in understanding the risk factors of SLE-ONFH and it also was beneficial for clinicians to take early interventions to prevent or slow the progression of ONFH in female SLE patients. Besides, a prediction model was developed and validated to provide an insightful and applicable tool for physicians to predict the risk of ONFH in female SLE patients.

## 2 Methods

### 2.1 Study design and patients

Clinical records of female SLE patients who fulfilled the American College of Rheumatology 1997 revised classification criteria for SLE (16) in The First Affiliated Hospital of Henan University of Chinese Medicine were retrospectively analyzed from January 2013 to December 2022 using an the electronic medical record database. All patients were divided into ONFH and non-ONFH groups based on the presence or absence of ONFH. The diagnosis of ONFH was based on the clinical manifestation and radiographic evidence including X-ray, computed tomography (CT), or magnetic resonance imaging (MRI) (17, 18). The following patients were exclude: (1) who had ONFH prior to being diagnosed with SLE; (2) ONFH caused by alcohol misuse, trauma, or the use of oral contraceptives; (3) who suffered from malignancy, pregnancy, or any other immune system diseases like rheumatoid arthritis; (4) did not have crucial initial data. Written informed consent was not required as this was a retrospective study utilizing clinical history data. The study has been approved by the Medical Ethics Committee of the First Affiliated Hospital of Henan University of Chinese Medicine (NO. 2023HL-285).

### 2.2 Data collection

Medical records for all patients were collected by experienced researchers, which included demographic data, clinical features, laboratory results, and previous treatments. Demographic data included age at SLE onset, disease duration at SLE onset, and major comorbidities including hypertension, osteoporosis, diabetes and hyperlipidemia. All of those complications occurred after the diagnosis of SLE. The following clinical characteristics including fever, skin rash, alopecia, oral ulcers, Raynaud's phenomenon, arthritis, serositis, lupus nephritis (LN), neuropsychiatric lupus, respiratory system involvement, gastrointestinal symptoms, hematological disorder, Sjögren's syndrome and menstrual abnormalities were documented at the initial hospitalization. Menstrual abnormalities (19) were defined as: 1) menstrual cycle frequency: frequent (<21 days), infrequent (>35 days), amenorrhea; 2) menstrual cycle regularity: irregular (≥7 days); 3) prolonged or shortened menstrual periods; 4) light menstrual bleeding or heavy menstrual bleeding; 5) blood stasis, and dysmenorrhea. Besides, disease activity was evaluated using the Disease Activity Index 2000 (SLEDAI-2K). At the time of diagnosis, laboratory records including routine blood analyses, lipid profile, complement levels were recorded. Autoantibody profiles were also documented including anti-double stranded DNA antibody (anti-dsDNA), anti-Smith anti-body (anti-Sm), anti-ribonucleoprotein antibody (anti-RNP), anti-ribosomal ribonucleoprotein antibody (anti-rRNP), anti-Sjogren Syndrome A antibody (anti-SSA), anti-Sjogren Syndrome B antibody (anti-SSB), anti-histone antibody (AHA), Anti-nucleosome antibody (anti-AnuA), and perinuclear anti-neutrophil cytoplasmic antibody (P-ANCA). The treatment information of GC exposure was documented, including whether or not receiving pulses of intravenous methylprednisolone (pulse MP, typically ranging from 125 to 1000 mg/day for 3-5 days) and the largest daily dose of GC that was converted to an equivalent amount of prednisolone according to the ratio of methylprednisolone: prednisone 4 to 5 (20). The immunosuppressive treatments included cyclophosphamide (CYC), methotrexate (MTX), mycophenolate mofetil (MMF), cyclosporine A (CsA), and tacrolimus (TAC). In addition, the use of biologics (belimumab or rituximab) and hydroxychloroquine (HCQ) were also collected.

### 2.3 Statistical analysis

For continuous variables adhering to a normal distribution, the mean ± standard deviation (SD) was used, whereas for non-normally distributed variables, the median along with the interquartile range (IQR) was used. For contrasting the two groups, either the t-test or the Mann–Whitney U test was employed. Variables of a categorical nature were displayed in terms of frequency (percentages) and underwent analysis through either the *χ^2^* test or Fisher's exact test. LASSO regression was utilized to optimize the screening variables related to ONFH in SLE patients, with the independent predictors subsequently identified through multivariate logistic regression analysis. Subsequently, the rms package was used to create the nomogram, relying on the independent predictors. The model's ability to differentiate was assessed by measuring the areas beneath the receiver operating characteristic curve (AUROC). To evaluate the calibration of the model, both the calibration curve and the Hosmer-Lemeshow goodness of fit test were employed. Decision curve analysis (DCA) was used to assess clinically effective.

All Statistical analyses were performed with IBM SPSS (version 25.0) and R software (version 4.3.2.) with rms, ramd, pROC, and glmnet package. The difference was considered statistically significant at *P* < 0.05.

## 3 Results

### 3.1 Study population

Of the 1034 patients initially identified with SLE at our hospital from January 2013 to December 2022, 241 were excluded, and 793 female SLE patients were ultimately included in this study. Among 793 female SLE patients, 87 patients developed ONFH, representing a prevalence rate of 10.9%.

### 3.2 Characteristics of ONFH in female SLE patients

The mean age at ONFH onset was (35.46 ± 13.08) years old. Among the 87 SLE-ONFH patients, 78 (89.7%) patients were reported with the symptom of pain or/and limited movement and 9 (10.3%) patients were reported asymptom. Besides, mostly patients (73.6%) had bilaterally involved, while 24(27.4%)had combined with knee joints (12.6%) or shoulders (2.3%) affected. Most patients were diagnosed ONFH via MRI (64.3%) (**Table 1**).

**Table 1:** Characteristics of ONFH in female SLE patients.

Clinical features	
Age at ONFH (years)	35.46 ± 13.08
Joint pain or / and limited movement	78 (89.7%)
Asymptom	9 (10.3%)
Location of ONFH	
Bilateral femoral heads	64 (73.6%)
Unilateral femoral head	24 (27.4%)
Combined with other lesion sites of ON	
Knee	11 (12.6%)
Shoulder	2 (2.3%)
Diagnostic tools	
MRI	39 (44.8%)
CT	10 (11.5%)
DR	21 (24.2%)
MRI+DR	17 (19.5%)

### 3.3 Comparison of demographic information, clinical features, laboratory findings, and treatment histories of SLE patients with and without ONFH

In the demographic information, there were significant difference in disease duration (3.00 (0.50, 7.00) *vs*. 6.00 (2.00, 13.00), *P* < 0.01) and osteoporosis rates (17.2% *vs*. 5.8%, *P* < 0.01) between the groups. In the clinical features, 35.8% of patients with menstrual abnormalities. Significant differences in the frequency of arthritis (67.8% *vs*. 55.9%, *P* = 0.034), respiratory symptoms (51.70% *vs*. 29.60%, *P* < 0.01), gastrointestinal symptoms (37.9% *vs*. 23.8%, *P* < 0.01), LN (59.80% *vs*. 42.80%, *P* < 0.01), Sjögren's syndrome (19.5% *vs*. 4.4%, *P* < 0.01), and menstrual abnormalities (64.40% *vs*. 32.3%, *P* < 0.01) were noted between the ONFH and non-ONFH groups. Additionally, the score of SLEDAI-2K was higher in the ONFH group compared to the non-ONFH group (14.10 ± 0.53 *vs*. 11.36 ± 0.18, *P* < 0.01). The comparison of laboratory findings between the ONFH and non-ONFH groups revealed a significantly higher rate of positive anti-RNP in the ONFH group (41.4% *vs*. 26.3%, *P* < 0.01). In the previous treatment strategies, the ONFH group showed a significantly higher median daily GC dose (60.00 *vs*. 50.00, *P* < 0.01) and a higher proportion of patients receiving treatment of pulse MP, CYC, MMF, and biologics compared to the non-ONFH group (21.8% *vs*. 13.6%, *P* = 0.039, 41.4% *vs.* 19.3%, *P* < 0.01; 50.6% *vs.* 33.7%, *P* = 0.002; 13.83% *vs.* 6.1%, *P* = 0.008, respectively) (**Table 2**).

**Table 2:** Comparison of demographic characters, clinical features, laboratory data and treatment history of total SLE patients between ONFH group and non-ONFH group.

Patients’ characteristics	Total (N=793)	ONFH group (N=87)	non-ONFH group (N=706)	*P*-value
demographic characters				
Disease duration (years)*	3.00 (0.50, 7.00)	6.00 (2.00, 13.00)	2.00 (0.50, 7.00)	<0.01
Age at SLE onset (years)	31.42 ± 12.76	30.11 ± 11.60	31.58 ± 12.89	0.314
Residence				0.776
Rural	376 (47.4%)	40 (46.0%)	336 (47.6%)	
Urban	417 (52.6%)	47 (54.0%)	370 (52.4%)	
Family history of SLE	7 (0.9%)	6 (0.8%)	1 (1.1%)	0.778
Comorbidities				
Hypertension	130 (16.4%)	14 (16.1%)	116 (16.4%)	0.936
Diabetes	30 (3.8%)	4 (4.6%)	26 (3.7%)	0.673
Osteoporosis*	56 (7.1%)	15 (17.2%)	41 (5.8%)	<0.01
Hyperlipidemia	350 (44.19%)	46 (52.9%)	304 (43.1%)	0.082
Clinical features				
Fever	420 (53.00%)	50 (57.50%)	370 (52.40%)	0.372
Skin rash	506 (63.80%)	59 (67.80%)	447 (63.30%)	0.41
Alopecia	213 (26.9%)	27 (31.0%)	186 (26.3%)	0.352
Oral ulcers	113 (14.2%)	14 (16.1%)	99 (14.0%)	0.602
Raynaud's phenomenon	136 (17.2%)	18 (20.7%)	118 (16.7%)	0.353
Arthritis*	454 (57.3%)	59 (67.8%)	395 (55.9%)	0.034
Serositis	79 (10.0%)	9 (10.30%)	70 (9.90%)	0.899
Respiratory system*	254 (32.00%)	45 (51.70%)	209 (29.60%)	<0.01
Gastrointestinal symptoms*	201 (25.3%)	33 (37.9%)	168 (23.8%)	0.004
lupus nephritis*	354 (44.60%)	52 (59.80%)	302 (42.80%)	0.003
Hematological disorder	582 (73.40%)	62 (71.3%)	520 (73.70%)	0.634
Neuropsychiatric lupus	36 (4.50%)	6 (6.9%)	30 (4.20%)	0.397
Sjögren's syndrome*	48 (6.1%)	17 (19.5%)	31 (4.4%)	<0.01
Menstrual abnormalities*	284 (35.8%)	56 (64.40%)	228 (32.3%)	<0.01
SLADAI-2K*	11.66 ± 4.95	14.10 ± 0.53	11.36 ± 0.18	<0.01
Laboratory findings				
Low C3	443 (55.9%)	41 (47.1%)	402 (56.9%)	0.082
Low C4	325 (41.0%)	38 (43.7%)	287 (40.7%)	0.588
Anti-dsDNA (+)	405 (51.1%)	45 (51.7%)	360 (51.0%)	0.897
Anti-Sm (+)	261 (32.9%)	29 (33.3%)	232 (32.9%)	0.93
Anti-RNP (+)*	222 (28.0%)	36 (41.4%)	186 (26.3%)	0.003
Anti-rRNP (+)	483 (60.9%)	50 (57.5%)	433 (61.3%)	0.486
Anti-SSA (+)	172 (21.7%)	13 (14.9%)	159 (22.5%)	0.106
Anti-SSB (+)	227 (28.6%)	20 (23.0%)	207 (29.3%)	0.218
Anti-AHA (+)	242 (30.5%)	31 (35.6%)	211 (29.9%)	0.272
Anti-AnuA (+)	232 (29.3%)	24 (27.6%)	208 (29.5%)	0.717
P-ANCA (+)	271 (34.2%)	33(37.9%)	238 (33.7%)	0.434
Treatments				
largest daily GC dose (mg)*	50.00 (30.00, 60.00)	60.00 (40.00, 75.00)	50.00 (30.00, 60.00)	<0.01
Pulse MP*	115 (14.5%)	19 (21.8%)	96 (13.6%)	0.039
CYC*	172 (21.7%)	36 (41.4%)	136 (19.3%)	<0.01
MMF*	282 (35.6%)	44 (50.6%)	238 (33.7%)	0.002
CsA	28 (3.5%)	4 (4.6%)	24 (3.4%)	0.792
TAC	33 (4.2%)	7 (8.0%)	26 (3.7%)	0.101
MTX	54 (6.8%)	6 (6.9%)	48 (6.8%)	0.973
HCQ	658 (83.0%)	69 (79.3%)	589 (83.4%)	0.335
Biologics*	55 (6.9%)	12 (13.83%)	43 (6.1%)	0.008

Data is presented with median (IQR), mean ± SD, or absolute count (%). Respiratory system involvement included pulmonary infection, bronchitis, pneumonia, and pulmonary interstitial fibrosis. Gastrointestinal symptoms included abdominal distension, abdominal pain, nausea, vomiting, diarrhea and anorexia. Hematological disorder include leucopenia (white blood cell count <4.0×10^9^/L) or thrombocytopenia (platelet count <100×10^9^/L). Low C3 or low C4 was defined as C3 < 0.85 g/L or C4 < 0.14 g/L. (+), positive. Largest daily GC dose was converted to an equivalent amount of prednisolone. Pulse MP, pulses of intravenous methylprednisolone. CYC, cyclophosphamide. MMF, mycophenolate mofetil. CsA, cyclosporine A. TAC, tacrolimus. MTX, methotrexate. HCQ, hydroxychloroquine. Biologics, belimumab or rituximab. * was represented the meaning of *P* < 0.05 or *P* < 0.01.

### 3.4 Development of a nomogram for predicting ONFH in female SLE patients

**Figure 1:**
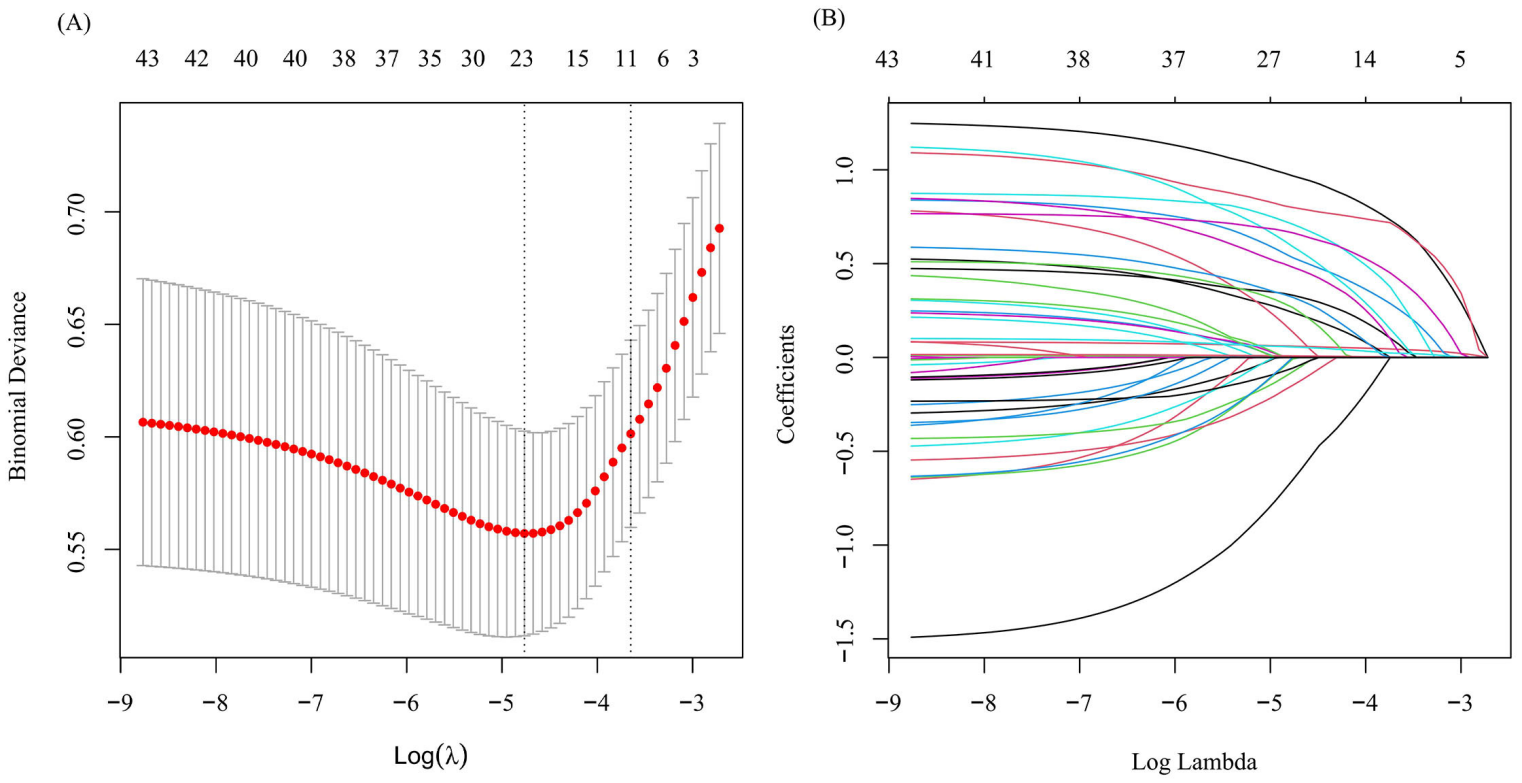
Optimize the screening variables by the LASSO regression. **(A)** LASSO cross validation curve to identify the optimal lambda through 10-fold cross-validation. **(B)** LASSO coefficient path diagram.

**Figure 2:**
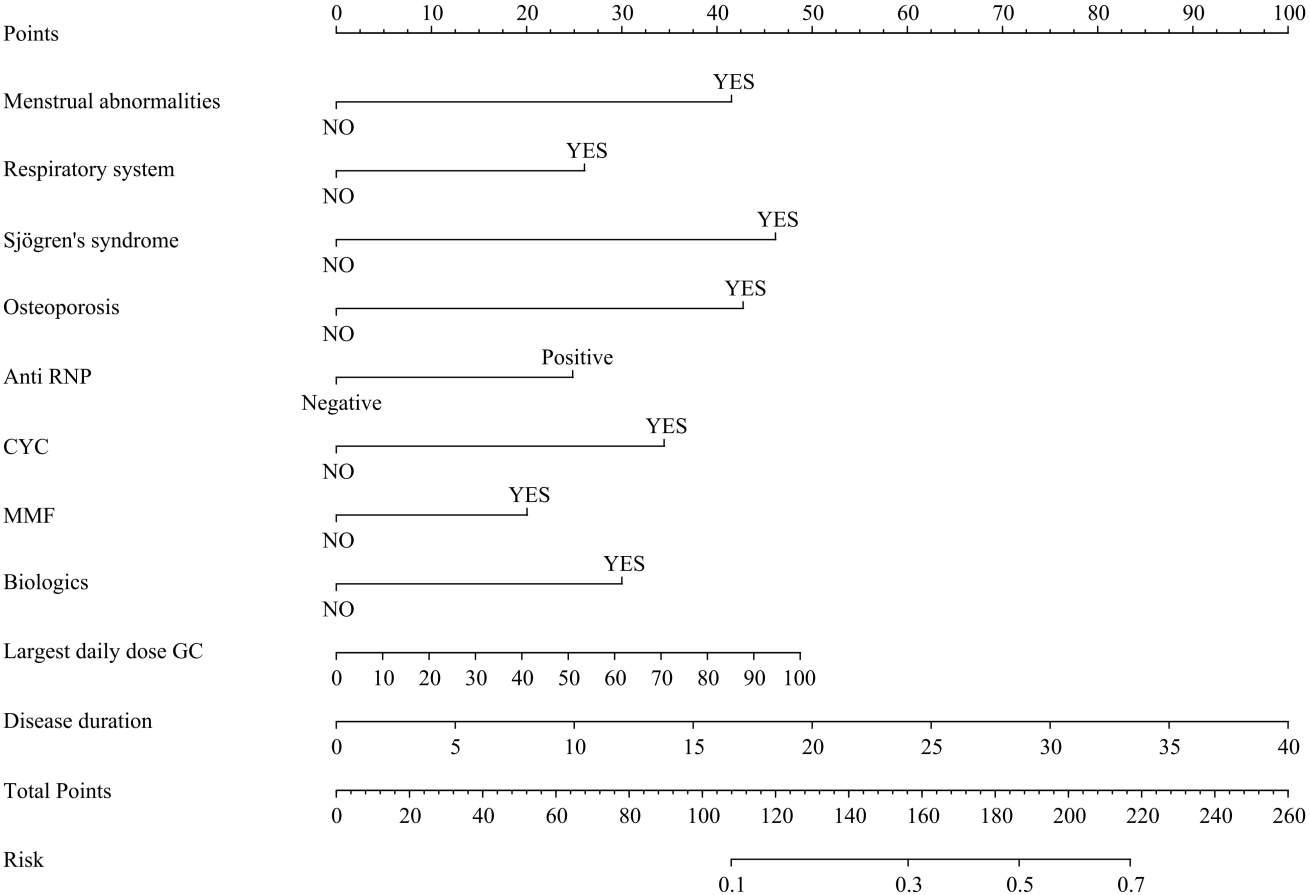
Nomogram for predicting the risk of ONFH in female SLE patients.

Considering the inter-dependencies and multi-collinearity among the included variables, LASSO regression analysis was utilized to identify predictive variables. With the optimum λ of 0.0260, eleven significant predictive variables with nonzero coefficients were identified through 10-fold cross-validation (**Figure 1**). Whether SLE patients have concurrent ONFH (no = 0, yes = 1) was used as the dependent variable, and eleven variables including disease duration at SLE onset (continuous variables, original value), respiratory involvement (no = 0, yes = 1), menstrual abnormalities (no = 0, yes = 1), Sjögren's syndrome (no = 0, yes = 1), osteoporosis (no = 0, yes = 1)、Anti-RNP (negative = 0, positive = 1), CYC (no = 0, yes = 1), MMF (no = 0, yes = 1), biologics (no = 0, yes = 1), SLADAI-2K (continuous variables, original value), and the largest daily GC dose (continuous variables, original value) screened by LASSO regression were used as independent variables. Multivariate logistic analysis is then performed using stepwise backward to identify independent risk factors linked to SLE-ONFH. Results revealed that ten variables—disease duration at SLE onset, respiratory involvement, menstrual abnormalities, Sjögren's syndrome, osteoporosis, Anti-RNP, CYC, MMF, biologics, and the largest daily GC dose—were significant risk factors for SLE-ONFH (**Table 3**). A nomogram (**Figure 2**) was developed to forecast ONFH likelihood in female SLE patients, using ten independent predictors that include disease duration at SLE onset, respiratory involvement, menstrual abnormalities, Sjögren's syndrome, osteoporosis, Anti-RNP, CYC, MMF, biologics, and the highest daily GC dose. The process of the multiple logistic regression analysis was visualized by the nomogram that can facilitate the computation of scores for each independent risk factor and the total score, predicting the likelihood of ONFH in female SLE patients.

**Table 3:** Multivariate logistic regression analysis for risk factors of SLE-ONFH.

Variables	β	SE	Wald	*P*-value	OR	*95%CI*
Menstrual abnormalities*	1.16	0.264	19.359	<0.001	3.189	1.903-5.347
Disease Duration*	0.07	0.017	15.984	<0.001	1.072	1.036-1.11
Respiratory system*	0.728	0.26	7.874	0.005	2.072	1.246-3.446
Sjögren's syndrome*	1.289	0.381	11.452	0.001	3.629	1.72-7.656
Osteoporosis*	1.194	0.398	9.013	0.003	3.3	1.514-7.197
Anti-RNP*	0.694	0.271	6.567	0.01	2.001	1.177-3.402
CTX*	0.962	0.276	12.195	<0.001	2.617	1.525-4.492
MMF*	0.56	0.264	4.504	0.034	1.75	1.044-2.935
Biologics*	0.838	0.421	3.967	0.046	2.312	1.013-5.275
Largest daily GC dose*	0.014	0.005	7.632	0.006	1.014	1.004-1.024

* was represented the meaning of *P* < 0.05 or *P* < 0.01.

### 3.5 Validation and calibration of the nomogram

**Figure 3:**
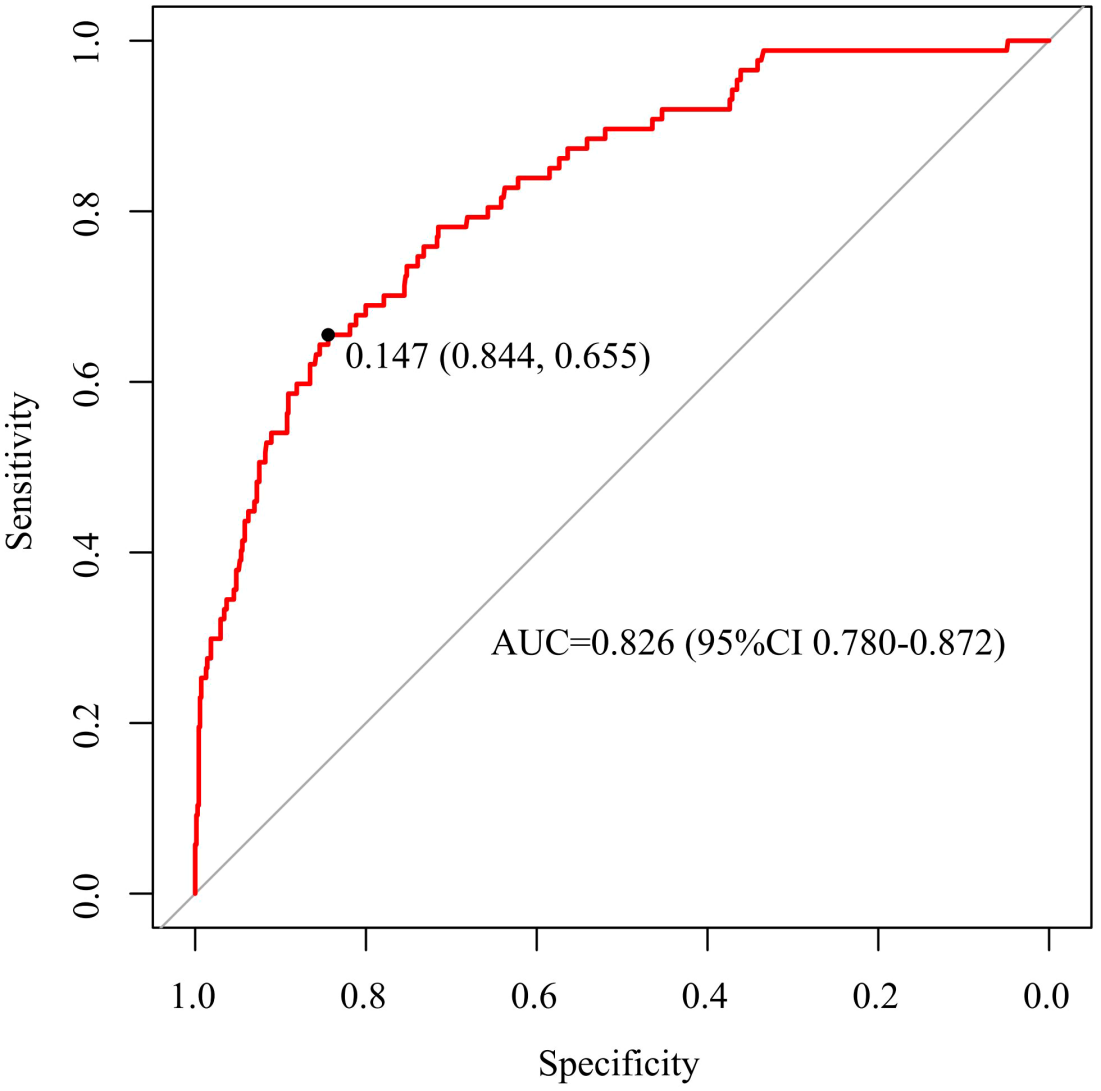
ROC curve of nomogram prediction model.

**Figure 4:**
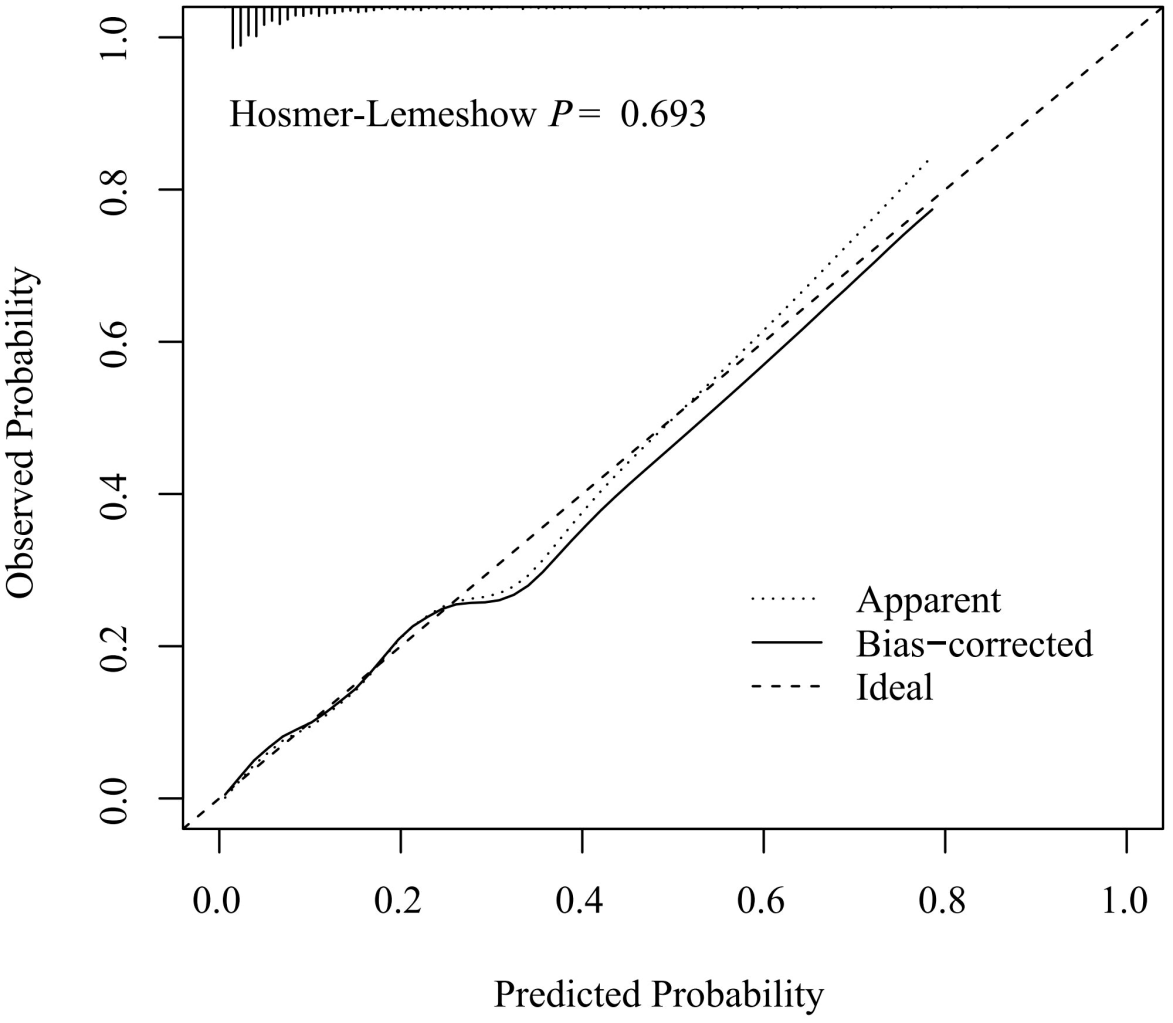
Calibration curve of nomogram prediction model.

The discriminative capacity of nomogram model was assessed through ROC analysis. The AUC of nomogram model was 0.826 (*95%CI*: 0.780-0.872), with an optimal threshold of 0.147 (sensitivity 0.844, specificity 0.655), demonstrating its strong predictive ability for ONFH occurrence in female SLE patients (**Figure 3**). A calibration curve and Hosmer-Lemeshow test were utilized to assess the calibration of the nomogram model. As depicted in **Figure 4**, the calibration curve of model was nearly matched the ideal one and Hosmer-Lemeshow test showed that the model was well calibrated (*χ^2^* = 5.589, *P* = 0.693), suggesting a strong correlation between the observed and actual outcomes.

### 3.6 Clinically effective of the nomogram

**Figure 5:**
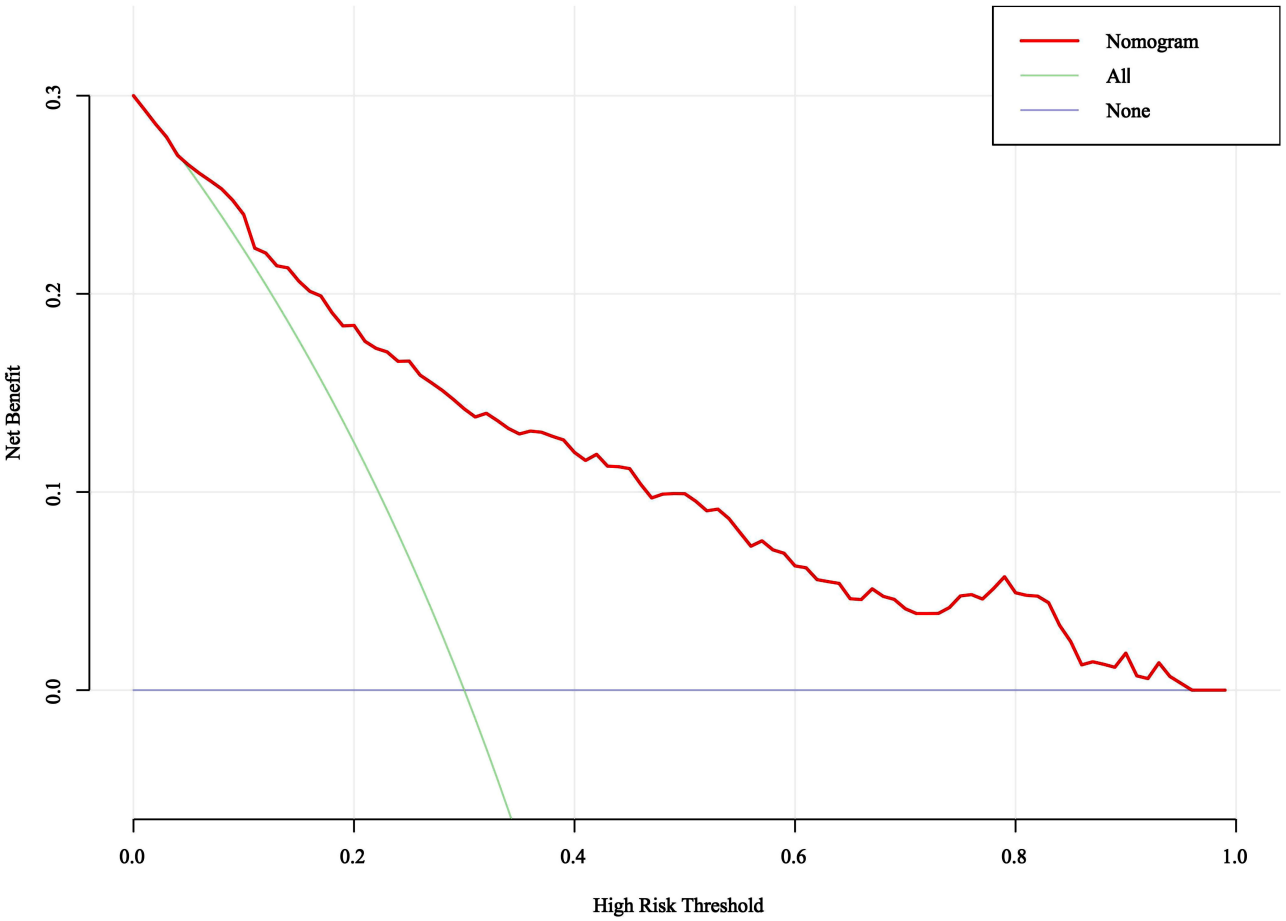
DCA analysis of the prediction model.

DCA was used to evaluate the clinical validity of the nomogram model. As depicted in **Figure 5**, when the threshold probabilities ranged from 0.05 to 0.95, the nomogram model demonstrated higher net benefits than all intervention and no intervention, showing that it has certain application in clinical practice.

### 3.7 Subgroup analysis

In order to further understand the impact of GC dosage and different treatment therapies on ONFH, we divided it into groups with largest daily GC dose < 50mg and ≥ 50mg according to the median of the largest daily GC dose. Meanwhile, based on different combination therapy strategies, we divided them into GC combined with immunosuppressants group, GC combined with biologics group, and GC combined with immunosuppressants and biologics group. Compared to the non-ONFH group, the ONFH group showed a significantly higher proportion of patients receiving treatment of largest daily GC dose ≥ 50mg, GC combined with immunosuppressants and GC combined with immunosuppressants and biologics (73.6% *vs*. 56.2%, *P* = 0.002, 64.4% *vs*. 51.0%, *P* < 0.018; 11.5% *vs*. 4.0%, *P* = 0.002, respectively), while a lower proportion of largest daily GC dose <50mg (26.4% *vs*. 43.8%, *P* = 0.002). (**Table 4**)

**Table 4:** Grouping situation of disease duration, age at SLE onset, largest daily GC dose and combination therapy.

	Total (N=793)	ONFH group (N=87)	non-ONFH group (N=706)	*P*-value
Disease duration (years)				
<3*	395 (49.8%)	26 (29.9%)	369 (52.3%)	<0.01
≥3*	398 (50.2%)	61 (70.1%)	337 (47.7%)	<0.01
Age at SLE onset				
≤18	132 (16.6%)	15 (17.2%)	117 (16.6%)	0.874
18-49	577 (72.8%)	67 (77.0%)	510 (72.2%)	0.345
≥49	84 (10.6%)	5 (5.8%)	79 (11.2%)	0.120
Largest daily GC dose (mg)				
<50*	332 (41.9%)	23 (26.4%)	309 (43.8%)	0.002
≥50*	461 (58.1%)	64 (73.6%)	397 (56.2%)	0.002
Combination therapy				
A*	416 (52.5%)	56 (64.4%)	360 (51.0%)	0.018
B	17 (2.1%)	2 (2.3%)	15 (2.1%)	0.916
C*	38 (4.8%)	10 (11.5%)	28 (4.0%)	0.002

A: GC combined with immunosuppressants group. B: GC combined with biologics group. C: GC combined with immunosuppressants and biologics group. * was represented the meaning of *P* < 0.05 or *P* < 0.01.

In addition, we conducted subgroup analysis in age at SLE onset, disease duration, largest daily GC dose, and combination therapy by using LASSO regressions and multivariate logistic regression analysis to explore the effect of menstrual abnormalities on the occurrence of ONFH in SLE. The results showed that, compared with disease duration of ≥3 years, the SLE patients with menstrual abnormalities in disease duration of < 3 years had a greater risk of developing ONFH and the highest risk increase was about 13.277 times. The risk of ONFH in SLE patients with menstrual abnormalities is greater in age at SLE onset of ≤18 and between 18-49 years old groups, especially in female patients younger than 18 years old. At the same time, SLE patients with menstrual abnormalities had a greater risk of developing ONFH in those using largest daily GC dose of ≥50 mg and GC combined with immunosuppressant groups. (**Table 5**)

**Table 5:** Subgroup analyses of the effect of menstrual abnormalities on ONFH in disease duration, age at SLE onset, largest daily GC dose and combination therapy.

	β	Wald	*P*-value	OR	95%CI
Disease duration (years)					
<3*	1.671	10.988	0.001	5.316	1.980-14.277
≥3*	1.375	16.658	<0.01	3.954	2.043-7.651
Age at SLE onset					
≤18*	2.634	8.14	0.004	13.924	2.281-85.114
18-49*	1.026	10.427	0.01	2.789	1.496-5.197
≥49			NA		
Largest daily GC dose (mg)					
<50			NA		
≥50*	1.507	18.885	<0.01	4.512	2.287-8.904
Combination therapy					
A*	1.228	13.696	<0.01	3.414	1.782-6.542
B			NA		
C			NA		

A: GC combined with immunosuppressants group. B: GC combined with biologics group. C: GC combined with immunosuppressants and biologics group. * was represented the meaning of *P* < 0.05 or *P* < 0.01.

## 4 Discussion

The pathogenesis of ONFH remains partially understood. ONFH is characterized by bone death, which may stem from lipid metabolism disorders, vascular coagulation disorders, apoptosis, autophagy, genetic factors, and other blood supply-related issues (21, 22). SLE is an autoimmune disease whose main pathological change is vasculitis. Terminal vasculitis of the femoral head can lead to vascular endothelial cell proliferation, perivascular inflammatory cell infiltration and embolism, while the collateral circulation is difficult to establish, which ultimately leads to bone ischemic necrosis (23). To date, numerous studies have evaluated the clinical features and risk factors of ONFH in SLE patients (24–26). However, there are fewer studies addressing these characteristics in female patients, especially those with menstrual abnormalities, and there is a lack of risk prediction models to predict the development of ONFH in patients with SLE. In our study, the risk factors for ONFH in female SLE patients were screened by LASSO regression and multivariate logistic regression and a nomogram model to predict ONFH occurrence was developed. LASSO regression analysis can enhance the stability of the model by penalizing the coefficients of all variables in the regression so that the coefficients of the relatively unimportant independent variables become zero. Nomogram prediction model can present complex regression equations as simple and easy-to-understand visual graphs, which it is not only easy to understand and operate than traditional prediction model formulas, but also is widely used in the demonstration of clinical prediction models for disease risk factors and disease diagnosis (27, 28).. Finally, in our study, ten variables including disease duration, respiratory involvement, menstrual abnormalities, Sjögren's syndrome, osteoporosis, anti-RNP, CYC, MMF, biologics, and the highest daily GC dose were identified the risk factors of ONFH in female SLE patients. Then, a nomogram prediction model was established based on this ten independent predictors, which could easily identify the risk of ONFH in female SLE patients by collecting information on multiple variables on this nomogram to calculate a total score for each patient. The predictive performance of the nomogram was assessed using ROC analysis, calibration curves and DCA. The area under the ROC curve of the nomogram model was 0.826 (*95% CI*: 0.780–0.872) and its calibration for forecasting the occurrence of ONFH was good, which was nearly matched actual observation results (*χ* ^2^ = 5.589, *P* = 0.693). DCA showed that the use of nomogram prediction model had certain application in clinical practice when the threshold was 0.05 to 0.95. Therefore, based to this nomogram model, high-risk groups could be early and timely identified by clinical staff, and take early interventions to prevent or slow the progression of ONFH.

In line with previous studies (7, 29) our analysis revealed that the disease duration of SLE in ONFH group was longer compared to the non-ONFH group. An Egyptian retrospective study (7) revealed that a disease duration exceeding 5 years independently correlated with ON development in SLE patients. Tselios et al. (29) observed a gradual increase in ON incidence when SLE patients' disease duration surpassed 10 years. This may be attributed to the fact that prolonged SLE duration leads to more accumulated organ damage and increased cumulative exposure to drugs like GC and immunosuppressants. But in subgroup analyses, we found that the risk of ONFH in SLE patients with menstrual abnormalities is greater in disease duration of <3 years. The underlying mechanisms still need further study.

The SLADAI-2K is the global and reliable score index to comprehensively access the disease activity of SLE including central nerve, vascular, renal, and musculoskeletal damage (30, 31). Several clinical studies and meta-analyses have demonstrated that SLE patients exhibiting higher SLADAI-2K scores possessed a significantly heightened risk of ONFH (24, 32). This association may be attributable to increased disease activity and severity, which subsequently necessitates the administration of higher doses of GC for disease management. Although the ONFH group had a higher SLADAI-2K score in this study, the results of multivariate logistic analysis did not support that SLADAI-2K was one of the independent risk factors of SLE - ONFH.

As crucial medications for SLE treatment, GCs effectively control inflammatory factor release, exerting anti-inflammatory effects (33). However, long-term application of GC in large quantities can impair endothelial cells, enhance intravascular coagulation, induce apoptosis of bone cells, promote intraosseous adipocyte hypertrophy and fat conversion of red marrow, which may increase risk of developing ONFH through reducing blood flow, oxygen delivery and increasing bone marrow pressure (34, 35). At present, there is a widespread consensus that the incidence of ONFH in SLE patients is linked to GC therapy. However, there is still controversy over whether the risk of ONFH is increased by mean or cumulative GC dosage, average daily dosage, maximum daily dosage, and pulse MP with concurrent ONHF in SLE (36–39). In our study, compared with the non-ONFH group, the maximum daily dose of GC in the ONFH group was significantly higher, especially largest daily GC dose of ≥50 mg, indicating that the higher the daily dose of GC was closely related to the occurrence of ONFH, which is also consistent with a multi-center Chinese SLE cohort by Cheng et al. (40). A prospective study of Kallas et al. also determined that the risk of ONFH with a daily GC dose greater than 60mg was 10.12 times higher than those who received a daily dose less than 20 mg (39). But, our study did not identify pulse MP as a risk factor for ONFH in SLE. Our study also found that immunosuppressants, especially CYC and MMF, were risk factors of ONFH in SLE patients. What is more, ONFH group showed a significantly higher proportion of patients receiving treatment of GC combined with immunosuppressant, especially in SLE patients with menstrual abnormalities. Previous study also showed that GC plus immunosuppressants could increase the risk factor of the development of AVN in SLE patients (7). Immunosuppressants directly prevent cell division of preosteoblasts, reduce the number of osteoblasts on the bone surface and inhibit bone formation, which may lead to the development of ONFH (41). Despite several previous studies reporting an association between the use of immunosuppressants and ONFH in SLE patients (42–44), the underlying mechanism remains elusive. Since immunosuppressive agents have been recommended to SLE patients, particularly those with more serious disease manifestations or high disease activity (45), it is challenging to isolate the impact of the high disease activity or its complications on the development of ONFH. Furthermore, a Japanese retrospective study found that the incidence of ON in SLE patients decreased from the previous 41% to 26.4% because immunosuppressant agents reduced the dependence of SLE patients on GC (38). Therefore, the impact of immunosuppressants on ONFH warrants further scrutiny.

After the occurrence of ON, the necrotic tissues and cells in the femoral head release various cytokines and a large number of inflammatory cells that the inflammatory response is activated and the original immune balance is destroyed, which can lead to bone destruction greater than bone production, and ultimately makes the femoral head irreversibly to the collapse (46). While, in SLE patients, B cells play a central role by participating in the production of autoantibodies, antigen processing and presentation, recruitment of autoreactive T cells, interaction with antigen-presenting cells, and cytokine secretion, leading to inflammation and tissue damage, which may further exacerbate the progression of ONFH (47, 48). A research indicated that ONFH patients had varying degrees of B-cell subpopulation imbalance and differences in cytokine levels, including elevated activated B-cells and decreased memory B-cells (49). Concurrently, osteogenic-osteoblastic imbalance is a prominent characteristic during ONFH onset and progression (50), and OPG/RANK/RANKL is an important signaling pathway involved in bone metabolism (51). The significantly lower mRNA expression levels of RANKL and OPG genes in patients with primary SLE may be related to the abnormal expression of RANKLx and OPG genes due to the abnormal function of T and B cells, which in turn caused the reduction of bone mass in SLE patients (52). Meanwhile, under physiological conditions, B cells play a protective role by promoting OPG production and inhibiting osteoclast formation. However, in inflammatory environments, they secrete a large number of inflammatory factors such as RANKL and TNF-α to promote osteoclast differentiation and maturation, which promotes bone resorption (53). Rituximab and belimumab, as novel B-cell-targeted therapies, can effectively inhibit B-cell proliferation and differentiation, and have good efficacy in reducing SLE disease activity and inflammatory factors (54). Consequently, they are recommended as first-line drugs for SLE in the latest guideline (31). Surprisingly, we found that use of biologics (rituximab or belimumab) was an independent risk factor of ONFH in SLE, potentially attributable to the limited number of patients using these biologics. At present, the relationship between biologics and ONFH has not been reported. Further explorations are needed to investigate the underlying mechanisms.

SLE patients commonly experience damage to the skin, kidneys, nervous system, blood system, respiratory system, and digestive system. In previous studies, LN, gastrointestinal symptoms, and arthritis have been reported as one of the risk factors for ONFH (55). Although LN, gastrointestinal symptoms, and arthritis occurred more frequently in ONFH group, the results of multifactorial analysis suggested that them did not increase the risk of developing ONFH.

Our study found that respiratory system involvement and Sjögren's syndrome were risk factors for ONFH. SLE often involves the respiratory system and can lead to pulmonary infection, bronchitis, pneumonia, pulmonary interstitial fibrosis, pulmonary arterial hypertension, acute respiratory distress syndrome and other lung diseases (56, 57). Chen et al. (58) reported that pulmonary arterial hypertension was one of the risk factors for SLE-ONFH, which may be due to the combined effects of multiple factors resulting in vascular endothelial cell dysfunction. Besides, most SLE patients with respiratory system involvement were more likely to be treated with high-dose GC (59). Sjögren's syndrome is one of the common complications of SLE and is characterized by the triad of sicca symptoms, fatigue and musculoskeletal pain (60). A meta-analysis conducted by Nevskaya et al. showed that SLE patients with Sjögren's syndrome could increase the risk of ONFH (9). In addition, a study reviewed retrospectively the records of 868 SLE patients demonstrated that Sjögren's syndrome were higher incidence in SLE patients with ONFH (61).

Additionally, our study was the first found that menstrual abnormalities were risk factors for ONFH. Menstruation, originating from the cyclic shedding of the endometrium, is often regulated and influenced by the hypothalamic pituitary ovarian system (HPOA). Previous studies indicated that SLE patients were susceptible to irregular menstruation, potentially due to the disease itself and the adverse effects of immunosuppressants and GC on the ovaries (13–15). In SLE patients, long-term chronic inflammation and elevated cytokine levels disrupt HPOA function and various ovarian-targeting mechanisms (15). GC, especially high-dose GC, may cause ovarian dysfunction, menstrual disorders and even amenorrhea, which may be related to the inhibition of the HPOA system and alteration of circulating gonadotropin levels (62). Additionally, immunosuppressants, like CYC, can impair gonadal function in SLE patients, increasing the risk of amenorrhea and ovarian insufficiency (63). Notably, SLE disease itself, immunosuppressants and GC are also risk factors for the development of ONFH in SLE. In subgroup analyses, we found that the risk of ONFH in SLE patients with menstrual abnormalities is greater in age at SLE onset of ≤ 18 years old, disease duration of <3 years, largest daily GC dose of ≥50 mg and the therapy of GC combined with immunosuppressant. And possible mechanisms to explain the higher risk in age at SLE onset of ≤ 18 years old include estrogen changes that increase the procoagulant effects and the red to yellow marrow conversion that can increase susceptibility to ischemic injury during puberty (26). This also suggested that personalized treatment strategies to prevent ONFH needed to be taken in advance when encountering SLE patients with menstrual abnormalities who are young, have a short course of disease, and have high doses of GC or GC combined with immunosuppressant in the clinic. But there are fewer reports on the association between ONFH and menstrual abnormalities. Consequently, further investigation is required to elucidate the specific mechanisms.

Moreover, osteoporosis also presented a risk factor for ONFH in SLE. Osteoporosis and ONFH are both bone metabolic abnormalities complicating SLE, and both can be mediated by inflammation and involved by various cytokines (64). Osteoporosis creates high pressure in the bone, which leads to the reduction of bone trabeculae and their strength, resulting in microcirculatory disorders such as decreasing the number of microvessels in the bone and the permeability of the vascular wall, which in turn leads to the development of ONFH (65).

Among laboratory data in this study, only positive anti-RNP antibodies were found significantly associated with ONFH, which was in accordance with previous studies (40, 66). Anti-RNP antibodies are expressed in SLE patients and are closely linked to mixed connective tissue disease (MCTD) (67). Additionally, anti-RNP can upregulate adhesion molecule expression, impact endothelial cells, and boost pro-inflammatory cytokine production, which may promote the development of ONFH (68).

While our study was the first to identify menstrual abnormalities and biologics as risk factors for female SLE-ONFH and establish a nomogram model, several limitations needed to be acknowledged. Firstly, as a retrospective study, it may have inherent selection biases. Besides, the subjects of this study were only female patients in Chinese Han population and could limit the generalizability of research findings. Secondly, since our study was based on reviewing the clinical medical records and there may be other reasons for this such as patient changed medication and dosage themselves, we only included the largest daily GC dose and pulse MP as variables to be analyzed and the impact of the cumulative duration of GC use, the cumulative GC dose and the average GC dose on ONFH were not analyzed. In order to further understand the impact of GC on ONFH risk, we divided the largest daily GC dose range and conducted subgroup analysis. At the same time, we explored the impact of different treatment strategies on the occurrence of ONFH. But, in future studies, there is still a need for more detailed documentation of GC use, dosage increase and decrease. Another limitation of our study is lack of external validation to further assess the predictive efficacy of the model. And due to the small number of ONFH events, the dataset was not split proportionally into a modeling set and a validation set for modeling and validation, respectively. In the future, we will collect more sample data to externally validate this nomogram model, conduct prospective, multi-center, large-sample studies to improve the predictive accuracy and generalizability of the model and explore the mechanisms underlying the association between menstrual abnormalities or biologics and ONFH in female SLE patients.

In conclusion, this study found that disease duration of SLE onset, respiratory involvement, menstrual abnormalities, Sjögren's syndrome, osteoporosis, Anti-RNP, CYC, MMF, biologics and largest daily dose of GC are risk factors for ONFH in female SLE patients. Besides, the risk of ONFH in SLE patients with menstrual abnormalities is greater in age at SLE onset of ≤ 18 years old, disease duration of <3 years, largest daily GC dose of ≥50 mg and the therapy of GC combined with immunosuppressant. Meanwhile, a prediction model for the risk of ONFH in female SLE was established, which had good predictive ability and clinical utility that could provide decision-making basis for clinicians to make early and effective diagnosis.

## Data Availability

The raw data supporting the conclusions of this article will be made available by the authors, without undue reservation.

## References

[1] Kiriakidou M Ching CL . Systemic lupus erythematosus. Ann Intern Med. (2020) 172:ITC81–96. doi: 10.7326/AITC202006020 32479157

[2] Kaneko K Chen H Kaufman M Sverdlov I Stein EM Park-Min KH . Glucocorticoid-induced osteonecrosis in systemic lupus erythematosus patients. Clin Transl Med. (2021) 11:e526. doi: 10.1002/ctm2.526 34709753 PMC8506634

[3] Ersin M Demirel M Ekinci M Mert L Çetin Ç Artım Esen B . Symptomatic osteonecrosis of the hip and knee in patients with systemic lupus erythematosus: Prevalence, pattern, and comparison of natural course. Lupus. (2021) 30:1603–8. doi: 10.1177/09612033211031007 34259056

[4] Trent S Sicat CS Slover J . Femoral head osteonecrosis in systemic lupus erythematosus: total hip arthroplasty outcomes and considerations. JBJS Rev. (2021) 9:e20.00142. doi: 10.2106/JBJS.RVW.20.00142 33819202

[5] Calvo-Alén J McGwin G Toloza S Fernández M Roseman JM Bastian HM . Systemic lupus erythematosus in a multiethnic US cohort (LUMINA): XXIV. Cytotoxic treatment is an additional risk factor for the development of symptomatic osteonecrosis in lupus patients: results of a nested matched case-control study. Ann Rheum Dis. (2006) 65:785–90. doi: 10.1136/ard.2005.040428 PMC1798170 16269429

[6] Oinuma K Harada Y Nawata Y Takabayashi K Abe I Kamikawa K . Osteonecrosis in patients with systemic lupus erythematosus develops very early after starting high dose corticosteroid treatment. Ann Rheum Dis. (2001) 60:1145–8. doi: 10.1136/ard.60.12.1145 PMC1753447 11709458

[7] Moghazy AA Ibrahim AM . Predictors of avascular necrosis in a cohort of Egyptian systemic lupus erythematosus patients: retrospective two centers study. Curr Rheumatol Rev. (2022) 18:144–49. doi: 10.2174/1573397117666210907124242 34493196

[8] Hagiwara S Nakamura J Watanabe A Kishida S Ohtori S Omae T . Corticosteroids and low bone mineral density affect hip cartilage in systemic lupus erythematosus patients: Quantitative T2 mapping. J Magn Reson Imaging. (2015) 42:1524–31. doi: 10.1002/jmri.24953 26019059

[9] Nevskaya T Gamble MP Pope JE . A meta-analysis of avascular necrosis in systemic lupus erythematosus: prevalence and risk factors. Clin Exp Rheumatol. (2017) 35:700–10. 28240590

[10] Suetsugu H Kim K Yamamoto T Bang SY Sakamoto Y Shin JM . Novel susceptibility loci for steroid-associated osteonecrosis of the femoral head in systemic lupus erythematosus. Hum Mol Genet. (2022) 31:1082–95. doi: 10.1093/hmg/ddab306 PMC8976424 34850884

[11] Webber D Cao J Dominguez D Gladman DD Knight A Levy DM . Genetics of osteonecrosis in children and adults with systemic lupus erythematosus. Rheumatol (Oxford). (2023) 62:3205–12. doi: 10.1093/rheumatology/kead016 36651668

[12] Tian J Zhang D Yao X Huang Y Lu Q . Global epidemiology of systemic lupus erythematosus: a comprehensive systematic analysis and modelling study. Ann Rheum Dis. (2023) 82:351–6. doi: 10.1136/ard-2022-223035 PMC9933169 36241363

[13] Hou Y Jin J Luo L Zhong Y Peng Z Song Z . Menstrual irregularity, pregnancy outcomes, and birth outcomes in patients with systemic lupus erythematosus of childbearing age in China: a multicenter cross-sectional study. Chin Med J (Engl). (2023) 136:2886–8. doi: 10.1097/CM9.0000000000002559 PMC10686584 37185157

[14] Morales-Martínez FA Salas-Castro C García-Garza MR Valdés-Martínez O García-Luna SM Garza-Elizondo M . Evaluation of the ovarian reserve in women with systemic lupus erythematosus. J Family Reprod Health. (2021) 15:38–44. doi: 10.18502/jfrh.v15i1.6076 34429735 PMC8346742

[15] Giambalvo S Garaffoni C Silvagni E Furini F Rizzo R Govoni M . Factors associated with fertility abnormalities in women with systemic lupus erythematosus: a systematic review and meta-analysis. Autoimmun Rev. (2022) 21:103038. doi: 10.1016/j.autrev.2022.103038 34995765

[16] Hochberg MC . Updating the American College of Rheumatology revised criteria for the classification of systemic lupus erythematosus. Arthritis Rheum. (1997) 40:1725. doi: 10.1002/art.1780400928 9324032

[17] Kim J Lee SK Kim JY Kim JH . CT and MRI findings beyond the subchondral bone in osteonecrosis of the femoral head to distinguish between ARCO stages 2 and 3A. Eur Radiol. (2023) 33:4789–800. doi: 10.1007/s00330-023-09403-8 36640174

[18] Di Matteo A Smerilli G Cipolletta E Salaffi F De Angelis R Di Carlo M . Imaging of joint and soft tissue involvement in systemic lupus erythematosus. Curr Rheumatol Rep. (2021) 23:73. doi: 10.1007/s11926-021-01040-8 34269905 PMC8285327

[19] Gynecologic Endocrinology Subgroup Chinese Society of Obstetrics and Gynecology Chinese Medical Association . Guideline on diagnosis and treatment of abnormal uterine bleeding: 2022 revisions. Chin J Obstet Gynecol. (2022) 57:481–90. doi: 10.3760/cma.j.cn112141-20220421-00258 35902781

[20] Dörner T Furie R . Novel paradigms in systemic lupus erythematosus. Lancet. (2019) 393:2344–58. doi: 10.1016/S0140-6736(19)30546-X 31180031

[21] Chang C Greenspan A Gershwin ME . The pathogenesis, diagnosis and clinical manifestations of steroid-induced osteonecrosis. J Autoimmun. (2020) 110:102460. doi: 10.1016/j.jaut.2020.102460 32307211

[22] Chen Y Miao Y Liu K Xue F Zhu B Zhang C . Evolutionary course of the femoral head osteonecrosis: Histopathological - radiologic characteristics and clinical staging systems. J Orthop Translat. (2021) 32:28–40. doi: 10.1016/j.jot.2021.07.004 35591937 PMC9072800

[23] Zheng Y Zheng Z Zhang K Zhu P . Osteonecrosis in systemic lupus erythematosus: Systematic insight from the epidemiology, pathogenesis, diagnosis and management. Autoimmun Rev. (2022) 21:102992. doi: 10.1016/j.autrev.2021.102992 34793961

[24] Zhang K Zheng Y Jia J Ding J Wu Z . Systemic lupus erythematosus patients with high disease activity are associated with accelerated incidence of osteonecrosis: a systematic review and meta-analysis. Clin Rheumatol. (2018) 37:5–11. doi: 10.1007/s10067-017-3820-5 28948379

[25] Qijiao W Meng Z Jianwen L Shengli Z Fei G He L . Antiphospholipid antibodies and osteonecrosis in systemic lupus erythematosus: a meta-analysis. Expert Rev Clin Immunol. (2021) 17:923–32. doi: 10.1080/1744666X.2021.1925109 33956556

[26] Tsai HL Chang JW Lu JH Liu CS . Epidemiology and risk factors associated with avascular necrosis in patients with autoimmune diseases: a nationwide study. Korean J Intern Med. (2022) 37:864–76. doi: 10.3904/kjim.2020.098 PMC9271726 35236014

[27] Park SY . Nomogram: An analogue tool to deliver digital knowledge. J Thorac Cardiovasc Surg. (2018) 155:1793. doi: 10.1016/j.jtcvs.2017.12.107 29370910

[28] Zhang Z Wang J Wang H Qiu Y Zhu L Liu J . An easy-to-use AIHF-nomogram to predict advanced liver fibrosis in patients with autoimmune hepatitis. Front Immunol. (2023) 14:1130362. doi: 10.3389/fimmu.2023.1130362 37266419 PMC10229817

[29] Tselios K Gladman DD Touma Z Su J Anderson N Urowitz MB . Disease course patterns in systemic lupus erythematosus. Lupus. (2019) 28:114–22. doi: 10.1177/0961203318817132 30526328

[30] Gladman DD Ibañez D Urowitz MB . Systemic lupus erythematosus disease activity index 2000. J Rheumatol. (2002) 29:288–91. 11838846

[31] Fanouriakis A Kostopoulou M Andersen J Aringer M Arnaud L Bae SC . EULAR recommendations for the management of systemic lupus erythematosus: 2023 update. Ann Rheum Dis. (2024) 83:15–29. doi: 10.1136/ard-2023-224762 37827694

[32] Sekiya F Yamaji K Yang K Tsuda H Takasaki Y . Investigation of occurrence of osteonecrosis of the femoral head after increasing corticosteroids in patients with recurring systemic lupus erythematosus. Rheumatol Int. (2010) 30:1587–93. doi: 10.1007/s00296-009-1194-y 19809818

[33] Hardy RS Raza K Cooper MS . Therapeutic glucocorticoids: mechanisms of actions in rheumatic diseases. Nat Rev Rheumatol. (2020) 16:133–44. doi: 10.1038/s41584-020-0371-y 32034322

[34] Huang C Wen Z Niu J Lin S Wang W . Steroid-induced osteonecrosis of the femoral head: novel insight into the roles of bone endothelial cells in pathogenesis and treatment. Front Cell Dev Biol. (2021) 9:777697. doi: 10.3389/fcell.2021.777697 34917616 PMC8670327

[35] Motta F Timilsina S Gershwin ME Selmi C . Steroid-induced osteonecrosis. J Transl Autoimmun. (2022) 5:100168. doi: 10.1016/j.jtauto.2022.100168 36213422 PMC9535426

[36] Felson DT Anderson JJ . Across-study evaluation of association between steroid dose and bolus steroids and avascular necrosis of bone. Lancet. (1987) 1:902–6. doi: 10.1016/s0140-6736(87)92870-4 2882300

[37] Joo YB Sung YK Shim JS Kim JH Lee EK Lee HS . Prevalence, incidence, and associated factors of avascular necrosis in Korean patients with systemic lupus erythematosus: a nationwide epidemiologic study. Rheumatol Int. (2015) 35:879–86. doi: 10.1007/s00296-014-3147-3 25300729

[38] Nawata K Nakamura J Ikeda K Furuta S Nakajima H Ohtori S . Transitional changes in the incidence of osteonecrosis in systemic lupus erythematosus patients: focus on immunosuppressant agents and glucocorticoids. Rheumatol (Oxford). (2018) 57:844–9. doi: 10.1093/rheumatology/key009 29462407

[39] Kallas R Li J Petri M . Predictors of osteonecrosis in systemic lupus erythematosus: A prospective cohort study. Arthritis Care Res (Hoboken). (2022) 74:1122–32. doi: 10.1002/acr.24541 PMC10725725 33342072

[40] Cheng C Huang C Chen Z Zhan F Duan X Wang Y . Risk factors for avascular necrosis in patients with systemic lupus erythematosus: a multi-center cohort study of Chinese SLE Treatment and Research Group (CSTAR) Registry XXII. Arthritis Res Ther. (2023) 25:78. doi: 10.1186/s13075-023-03061-3 37173771 PMC10176939

[41] Zhao D Wang C Zhao Y Shu B Jia Y Liu S . Cyclophosphamide causes osteoporosis in C57BL/6 male mice: suppressive effects of cyclophosphamide on osteoblastogenesis and osteoclastogenesis. Oncotarget. (2017) 8:98163–83. doi: 10.18632/oncotarget.21000 PMC5716721 29228681

[42] Hussein S Suitner M Béland-Bonenfant S Baril-Dionne A Vandermeer B Santesso N . Monitoring of osteonecrosis in systemic lupus erythematosus: A systematic review and metaanalysis. J Rheumatol. (2018) 45:1462–76. doi: 10.3899/jrheum.170837 29961688

[43] Faezi ST Hoseinian AS Paragomi P Akbarian M Esfahanian F Gharibdoost F . Non-corticosteroid risk factors of symptomatic avascular necrosis of bone in systemic lupus erythematosus: A retrospective case-control study. Mod Rheumatol. (2015) 25:590–4. doi: 10.3109/14397595.2014.987366 25528860

[44] Lee J Kwok SK Jung SM Min HK Nam HC Seo JH . Osteonecrosis of the hip in Korean patients with systemic lupus erythematosus: risk factors and clinical outcome. Lupus. (2014) 23:39–45. doi: 10.1177/0961203313512880 24335586

[45] Fanouriakis A Kostopoulou M Alunno A Aringer M Bajema I Boletis JN . 2019 update of the EULAR recommendations for the management of systemic lupus erythematosus. Ann Rheum Dis. (2019) 78:736–45. doi: 10.1136/annrheumdis-2019-215089 30926722

[46] Ma J Ge J Gao F Wang B Yue D Sun W . The role of immune regulatory cells in nontraumatic osteonecrosis of the femoral head: A retrospective clinical study. BioMed Res Int. (2019) 2019:1302015. doi: 10.1155/2019/1302015 31828086 PMC6886356

[47] Ghobadinezhad F Ebrahimi N Mozaffari F Moradi N Beiranvand S Pournazari M . The emerging role of regulatory cell-based therapy in autoimmune disease. Front Immunol. (2022) 13:1075813. doi: 10.3389/fimmu.2022.1075813 36591309 PMC9795194

[48] Fasano S Milone A Nicoletti GF Isenberg DA Ciccia F . Precision medicine in systemic lupus erythematosus. Nat Rev Rheumatol. (2023) 19:331–42. doi: 10.1038/s41584-023-00948-y 37041269

[49] Zhang H Xiao F Liu Y Zhao D Shan Y Jiang Y . A higher frequency of peripheral blood activated B cells in patients with non-traumatic osteonecrosis of the femoral head. Int Immunopharmacol. (2014) 20:95–100. doi: 10.1016/j.intimp.2014.02.016 24583150

[50] Wang P Wang C Meng H Liu G Li H Gao J . The role of structural deterioration and biomechanical changes of the necrotic lesion in collapse mechanism of osteonecrosis of the femoral head. Orthop Surg. (2022) 14:831–9. doi: 10.1111/os.13277 PMC9087473 35445585

[51] Ono T Hayashi M Sasaki F Nakashima T . RANKL biology: bone metabolism, the immune system, and beyond. Inflammation Regener. (2020) 40:2. doi: 10.1186/s41232-019-0111-3 PMC7006158 32047573

[52] Adami G Fassio A Rossini M Caimmi C Giollo A Orsolini G . Osteoporosis in rheumatic diseases. Int J Mol Sci. (2019) 20:5867. doi: 10.3390/ijms20235867 31766755 PMC6928928

[53] Lee DSW Rojas OL Gommerman JL . B cell depletion therapies in autoimmune disease: advances and mechanistic insights. Nat Rev Drug Discovery. (2021) 20:179–99. doi: 10.1038/s41573-020-00092-2 PMC7737718 33324003

[54] Cheng H Zhang XY Yang HD Yu Z Yan CL Gao C . Efficacy and safety of belimumab/low-dose cyclophosphamide therapy in moderate-to-severe systemic lupus erythematosus. Front Immunol. (2022) 13:911730. doi: 10.3389/fimmu.2022.911730 35979351 PMC9376229

[55] Long Y Zhang S Zhao J You H Zhang L Li J . Risk of osteonecrosis in systemic lupus erythematosus: An 11-year Chinese single-center cohort study. Lupus. (2021) 30:1459–68. doi: 10.1177/09612033211021166 34082592

[56] Di Bartolomeo S Alunno A Carubbi F . Respiratory manifestations in systemic lupus erythematosus. Pharm (Basel). (2021) 14:276. doi: 10.3390/ph14030276 PMC8003168 33803847

[57] Li Y Qian J Dong X Zhao J Wang Q Wang Y . The prognosis and management of reclassified systemic lupus erythematosus associated pulmonary arterial hypertension according to 2022 ESC/ERS guidelines. Arthritis Res Ther. (2024) 26:109. doi: 10.1186/s13075-024-03338-1 38802957 PMC11129383

[58] Chen S Cai Q Xu Y Fu Q Feng Y Chen X . Associations between glucocorticoids, antiphospholipid antibodies and femur head necrosis in patients with SLE: a directed acyclic graph-based multicenter study. Ther Adv Musculoskelet Dis. (2021) 13:1759720x211002677. doi: 10.1177/1759720X211002677 PMC8010842 33854569

[59] Takahashi S Fukushima W Yamamoto T Iwamoto Y Kubo T Sugano N . Temporal trends in characteristics of newly diagnosed nontraumatic osteonecrosis of the femoral head from 1997 to 2011: A hospital-based sentinel monitoring system in Japan. J Epidemiol. (2015) 25:437–44. doi: 10.2188/jea.JE20140162 PMC4444498 25912097

[60] Cui Y Zhang H Wang Z Gong B Al-Ward H Deng Y . Exploring the shared molecular mechanisms between systemic lupus erythematosus and primary Sjögren's syndrome based on integrated bioinformatics and single-cell RNA-seq analysis. Front Immunol. (2023) 14:1212330. doi: 10.3389/fimmu.2023.1212330 37614232 PMC10442653

[61] Sayarlioglu M Yuzbasioglu N Inanc M Kamali S Cefle A Karaman O . Risk factors for avascular bone necrosis in patients with systemic lupus erythematosus. Rheumatol Int. (2012) 32:177–82. doi: 10.1007/s00296-010-1597-9 20711782

[62] de Medeiros SF Rodgers RJ Norman RJ . Adipocyte and steroidogenic cell cross-talk in polycystic ovary syndrome. Hum Reprod Update. (2021) 27:771–96. doi: 10.1093/humupd/dmab004 33764457

[63] Sharma SK Jain S Bahl P Potturi P Rathi M Naidu S . Ovarian dysfunction with moderate-dose intravenous cyclophosphamide (modified NIH regimen) and mycophenolate mofetil in young adults with severe lupus: a prospective cohort study. Arthritis Res Ther. (2020) 22:189. doi: 10.1186/s13075-020-02292-y 32799907 PMC7429750

[64] Azizieh F Raghupathy R Shehab D Al-Jarallah K Gupta R . Cytokine profiles in osteoporosis suggest a proresorptive bias. Menopause. (2017) 24:1057–64. doi: 10.1097/GME.0000000000000885 28609384

[65] Liu Y Cao L Hillengass J Delorme S Schlewitz G Govindarajan P . Quantitative assessment of microcirculation and diffusion in the bone marrow of osteoporotic rats using VCT, DCE-MRI, DW-MRI, and histology. Acta Radiol. (2013) 54:205–13. doi: 10.1258/ar.2012.120508 23319721

[66] Xiong J Wang G Xu T Liu R Yu S Wang Y . Anti-RNP antibody: A potential novel predictor for osteonecrosis in systemic lupus erythematosus. Front Med (Lausanne). (2022) 9:847875. doi: 10.3389/fmed.2022.847875 35479947 PMC9035537

[67] Wesner N Uruha A Suzuki S Mariampillai K Granger B Champtiaux N . Anti-RNP antibodies delineate a subgroup of myositis: A systematic retrospective study on 46 patients. Autoimmun Rev. (2020) 19:102465. doi: 10.1016/j.autrev.2020.10246 31918028

[68] Matsueda Y Arinuma Y Nagai T Hirohata S . Synergistic enhancement of production of proinflammatory cytokines of human peripheral blood monocytes by anti-Sm and anti-RNP antibodies. PloS One. (2018) 13:e0209282. doi: 10.1371/journal.pone.0209282 30571738 PMC6301657

